# Evidence of Associations Between Feto-Maternal Vitamin D Status, Cord Parathyroid Hormone and Bone-Specific Alkaline Phosphatase, and Newborn Whole Body Bone Mineral Content

**DOI:** 10.3390/nu4020068

**Published:** 2012-02-06

**Authors:** Daphna K. Dror, Janet C. King, Ellen B. Fung, Marta D. Van Loan, Erik R. Gertz, Lindsay H. Allen

**Affiliations:** 1 USDA, ARS, Western Human Nutrition Research Center, 430 W. Health Sciences Dr., Davis, CA 95616, USA; Email: marta.vanloan@ars.usda.gov (M.D.V.L.); erik.gertz@ars.usda.gov (E.R.G.); marta.vanloan@ars.usda.gov (L.H.A.); 2 Children’s Hospital & Research Center, 747 52nd St., Oakland, CA 94609, USA; Email: jking@chori.org (J.C.K.); efung@mail.cho.org (E.B.F.)

**Keywords:** infant, vitamin D, dual X-ray absorptiometry, bone mineral content

## Abstract

In spite of a high prevalence of vitamin D inadequacy in pregnant women and neonates, relationships among vitamin D status (25(OH)D), parathyroid hormone (PTH), bone specific alkaline phosphatase (BALP), and whole body bone mineral content (WBBMC) in the newborn are poorly characterized. The purpose of the present study was to investigate the relationships between maternal and cord 25(OH)D, PTH, BALP, and WBBMC in newborns in a multiethnic population in Oakland, California and to evaluate the predictive value of the biochemical indices as indicators of WBBMC. Maternal and cord blood were collected from 80 mother-infant pairs and infant WBBMC was measured by dual energy X-ray absorptiometry 8–21 days post-birth. Cord PTH and BALP were each inversely correlated with infant WBBMC (*r* = −0.28, *p* = 0.01 and *r* = −0.26, *p* = 0.02) and with cord 25(OH)D (*r* = −0.24, *p* = 0.03 and *r* = −0.34, *p* = 0.002), while cord 25(OH)D and unadjusted or weight-adjusted WBBMC were not significantly correlated with one other. In multivariate regression modeling, infant WBBMC was most strongly predicted by infant weight (*p* < 0.0001), while either PTH or BALP contributed modestly but significantly to the model (*p* = 0.006 and *p* = 0.03 respectively). Cord 25(OH)D was not a significant predictor of infant WBBMC. This study provides evidence of associations between feto-maternal 25(OH)D, cord PTH and BALP, and early infant WBBMC, though neither feto-maternal 25(OH)D nor the measured biochemical indices were suitable indicators of WBBMC.

## 1. Introduction

Vitamin D inadequacy is highly prevalent amongst pregnant women and neonates in diverse populations with possible implications on offspring bone health [1]. In teens and adults, parathyroid hormone (PTH) and bone specific alkaline phosphatase (BALP) are elevated in vitamin D deficiency and are associated with enhanced bone turnover [2,3]. However, the behavior of these biomarkers in the fetus and their relationship with vitamin D status and neonatal bone mineralization are poorly characterized.

Secondary hyperparathyroidism is a classic sign of vitamin D deficiency, where low ionized calcium concentrations trigger a sensor in the parathyroid glands to increase PTH secretion. Elevated PTH concentrations are associated with a decrease in bone mineralization and increase in bone resorption leading to osteomalacia in adults and rickets in children [4,5]. In the fetus, a hypercalcemic state is maintained by active transport of calcium across the placenta and PTH is suppressed, with a further decrease towards the end of gestation [6,7,8]. However, evidence from PTH-ablated mice reveals significantly decreased mineralization of the fetal cartilage matrix, suggesting a critical role of PTH in normal bone development [9]. 

BALP is a tetrameric membrane glycoprotein of osteoblasts that is released into the extracellular space following cleavage by phospholipases and is considered a biomarker of bone formation [10]. However, multiple studies have demonstrated an inverse association between BALP and bone mineral content (BMC) measured by dual X-ray absorptiometry (DXA) in various population groups [11,12,13], likely related to a concurrent and more pronounced increase in bone resorption [11]. Bone biomarkers including BALP increase during pregnancy regardless of vitamin D status and attain higher values in umbilical cord blood than maternal blood [14], possibly reflecting higher rates of bone turnover. 

Using a novel imaging technique to quantify intrauterine bone growth, the group of Mahon *et al.* demonstrated that maternal vitamin D status can influence fetal femoral development as early as 19 weeks gestation [15]. Intrauterine effects on fetal bone mineral accrual may have consequences on bone health later in life [14]. However, there are gaps in the understanding of whether and how vitamin D influences fetal bone development and whether markers of bone turnover in cord blood predict bone mineral content. The purpose of the present study was to investigate the relationships among maternal and cord vitamin D status, PTH, BALP, and whole body BMC (WBBMC) in newborns in a multiethnic population in Oakland, California and to evaluate the predictive value of the biochemical indices as indicators of neonatal WBBMC.

## 2. Subjects and Methods

The study was approved by the institutional review boards of the University of California, Davis, Children’s Hospital and Research Center, Oakland, and Alta Bates Medical Center, Berkeley. Recruitment took place between December 2006 and January 2008. No routine vitamin D screening was provided during antenatal care at the clinic or hospital.

Pregnant women were recruited from East Bay Perinatal Medical Associates in Oakland, California during a prenatal visit approximately one month before their due date. Women who were 18–45 years of age, planning to deliver at Alta Bates Medical Center, and carrying a singleton fetus were eligible to participate. Informed consent was obtained upon study enrollment. Average daily dietary intake of vitamin D was calculated from food frequency using food composition data from the United States Department of Agriculture National Nutrient Database [16]. In an interviewer-administered questionnaire, participants were asked to estimate frequency and quantity of the main dietary sources of vitamin D (fatty fish, milk, breakfast cereals, fortified orange juice, eggs, and meat) consumed in the month prior to the interview. Medical records were reviewed to ascertain pre- or early-pregnancy weight, length of gestation, pregnancy and delivery events, and infant birth weight. Pre-gestational maternal body mass index (BMI) was calculated from reported height and pre- or early-pregnancy weight recorded in the medical file. Race was self-reported.

Maternal venous blood was collected upon admission to the Alta Bates Medical Center Labor and Delivery Unit and cord blood collected immediately post delivery. Blood samples were kept at 4 °C until centrifuged and serum stored at −80 °C until analysis. Batched samples of serum 25(OH)D were assayed monthly at ARUP Laboratories (Salt Lake City, UT, USA) using the DiaSorin radioimmunoassay (DiaSorin Inc., Stillwater, MN, USA). An internal standard which had been assayed in duplicate in the laboratory of Bruce Hollis (Medical University of South Carolina, Charleston, SC, USA) was included with each batch and results were adjusted accordingly. BALP in a subset of cord serum samples was measured by ELISA (Quidel Corp, San Diego, CA, USA). Intact PTH was measured by chemiluminescent enzyme-labeled immunometric assay with the Immulite autoanalyzer (Diagnostic Products Corporation, Los Angeles, CA, USA).

Mothers and term-born infants (37–42 weeks gestation) returned for a follow-up visit at Children’s Hospital of Oakland CTSI Clinical Research Center 8–21 days post-birth. The infants’ length, weight, and head circumference were measured by research staff using the World Health Organization standardized protocol [17]. Weight was measured to the nearest gram using an infant digital scale (Seca 334, Seca Corp., Hamburg, Germany), length to the nearest 0.1 cm using an infant length board (Shorr Infant Polylength Measuring Board, Shorr Productions, Olney, MD, USA), and head circumference to the nearest 0.1 cm using nonstretchable tape (Shorr Productions, Olney, MD, USA). Bone mineral content was determined by dual-energy X-ray absorptiometry (DXA) conducted in duplicate using the Hologic Discovery A whole body infant software package version 12.6.1 (Hologic Inc., Waltham, MA, USA). Prior to imaging each infant was dressed only in a clean study-provided diaper and swaddled in a single cotton receiving blanket to restrict movement. If possible, infants were breast- or formula-fed and rocked to sleep immediately prior to the scan, and the scan with least movement artifact was selected for inclusion in analysis.

## 3. Statistical Analysis

Measurement of infant WBBMC by DXA in the present study was conducted as a secondary outcome in a comprehensive study estimating prevalence, predictors, and consequences of vitamin D status during pregnancy [18]. Of 275 women initially enrolled, cord blood samples from 80 infants with successful DXA scans were selected for measurement of BALP, allowing for detection of correlation ρ > 0.28 between study variables at α = 0.05 and power = 0.8. Infants included in this analysis did not differ significantly from those who underwent DXA scans but were not included or who were eligible but did not return for the DXA visit in gender, maternal age, body mass index, L*, parity, or maternal or cord vitamin D status.

Statistical analysis was performed using SAS 9.1 software (SAS Institute Inc., Cary, NC, USA). Data are presented as means ± SD. Normality was tested using the Shapiro-Wilk statistic and log transformations applied to non-normally distributed variables. Correlations between continuous variables were estimated using Pearson’s correlation coefficient. For purposes of data analysis, undetectable values were assigned as 2/3 the detection limit. This method is used to approximate the mean values of the tail of the distribution. Backward elimination was used in multivariate regression to build a predictive model for infant WBBMC. In an alternate multivariate model, WBBMC was adjusted for infant size using logarithmic analysis. For all tests, *p* < 0.05 was considered significant. 

## 4. Results

Cord 25(OH)D was inversely correlated with both cord PTH (*r* = −0.24, *p* = 0.03) and BALP (*r* = −0.34, *p* = 0.002) while cord PTH and BALP were positively correlated with one another (*r* = 0.27, *p* = 0.02). A positive correlation was found between maternal and cord 25(OH)D (*r* = 0.78, *p* < 0.0001) but not between maternal and cord PTH (Table 1). 

**Table 1:** Correlations between parameters (Pearson’s correlation coefficient and *p*-value).

	Cord 25(OH)D ^1^	Maternal PTH ^1^	Cord PTH ^1^	Cord BALP ^1^	Unadjusted WBBMC	Adjusted WBBMC ^2^
Maternal 25(OH)D	0.78	−0.44	−0.19	−0.26	NS	0.21
	<0.0001	<0.0001	0.09	0.02		0.06
Cord 25(OH)D ^1^	-	−0.43	−0.24	−0.34	NS	0.23
		<0.0001	0.03	0.002		0.04
Maternal PTH ^1^		-	NS	0.21	NS	NS
				0.07		
Cord PTH ^1^			-	0.27	−0.28	−0.29
				0.02	0.01	0.01
Cord BALP ^1^				-	−0.26	−0.21
					0.02	0.07

^1^ Log transformed to conform to normal distribution; ^2^ WBBMC/(infant weight^1.31).

**Figure 1:**
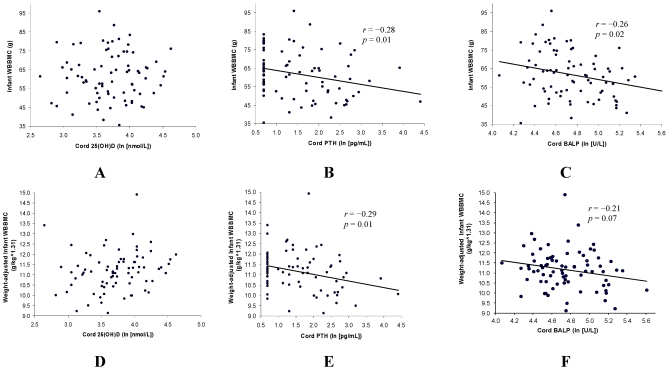
Associations of unadjusted (**A–C**) and weight-adjusted (**D–F**) infant whole body bone mineral content (WBBMC) with cord serum 25(OH)D, parathyroid hormone (PTH), and bone specific alkaline phosphatase (BALP).

Unadjusted infant WBBMC was inversely correlated with cord PTH (*r* = −0.28, *p* = 0.01) and BALP (*r* = −0.26, *p* = 0.02) but was not correlated with cord 25(OH)D (Figure 1). It was determined through logarithmic analysis that a size-neutral measurement of WBBMC was approximated by WBBMC/(weight^1.31). Adjusted WBBMC was inversely correlated with cord PTH (*r* = −0.29, *p* = 0.01), marginally inversely correlated with BALP (*r* = −0.21, *p* = 0.07), and was not correlated with cord 25(OH)D.

None of the measured indices differed significantly by infant sex or by use of maternal vitamin D supplements during pregnancy. Cord serum 25(OH)D was significantly higher, and maternal serum 25(OH)D marginally higher, in women whose total daily vitamin D intake was estimated to be >600 IU/day (*n* = 50, *p* = 0.0015 and *p* = 0.06 for cord and maternal values, respectively). PTH was undetectable in 30 (37.5%) of the cord samples, with no significant difference in proportion of undetectable PTH in subjects by race, sex, or cord serum 25(OH)D (≤ or >37.5 nmol/L, *p* > 0.05 for all). 

Compared with non-African Americans, African American infants had lower cord 25(OH)D (*p* = 0.004) and higher cord PTH (*p* = 0.009) and BALP (*p* = 0.04). Maternal 25(OH)D was also lower in African American compared with non-African American mothers (*p* = 0.04), but maternal PTH did not differ significantly by race. Neonatal unadjusted WBBMC did not differ by race, while weight-adjusted WBBMC was marginally lower in African American infants (*p* = 0.06) (Table 2). 

**Table 2:** Characteristics of African American and non–African American participants.

	African American (*n* = 40)		Non–African American (*n* = 40)		*p*–value
	Mean (SD) ^1^	Range	Mean (SD) ^1^	Range	
Gestational age at birth (weeks)	39.7 (1.6)	37.1–42.0	39.4 (1.1)	37.1–41.3	NS
Birth weight (g)	3429 (512)	2372–4532	3419 (526)	2290–4460	NS
Age at DXA scan (days)	12.8 (3.4)	8–21	13.4 (3.5)	9–21	NS
Maternal parity	2.1 (1.2)	1–5	1.8 (0.8)	1–3	NS
Maternal age (years)	25.4 (5.2)	18–39	28.3 (5.6)	18–38	0.02
Maternal BMI (kg/m^2^) ^1^	32.2 (29.6, 35.0)	18.8–49.9	30.9 (28.3, 33.7)	20.5–66.0	NS
Maternal total vitamin D intake (IU/days)	698 (246)	0–1326	684 (253)	184–1316	NS
Maternal 25(OH)D (nmol/L)	69.1 (26.1)	33.5–118.5	82.3 (30.3)	20.8–151.7	0.04
Cord 25(OH)D ^1,2^ (nmol/L)	36.0 (31.7, 40.8)	16.7–87.5	48.2 (41.4, 56.1)	14.1–157.5	0.004
Maternal PTH (pg/mL) ^1^	29.4 (24.9, 34.8)	9.5–66.1	29.3 (23.6, 36.4)	6.2–102.0	NS
Cord PTH (pg/mL) ^1,2^	6.1 (4.6, 8.0)	2.0–50.7	3.7 (2.8, 4.7)	2.0–82.5	0.009
Cord BALP (U/L) ^1^	123.9 (112.2, 137.1)	71.4–272.5	107.7 (98.4, 117.9)	58.3–197.8	0.04
WBBMC (g)	61.3 (13.1)	38.3–95.9	61.9 (12.1)	35.4–83.3	NS
WBBMC (g per (kg^1.31))	10.9 (0.9)	9.1–12.7	11.4 (1.0)	8.9–14.9	0.06
	*N*	%	*n*	%	*p*–value
Sex					NS
Male	22	55	18	45	
Female	18	45	22	55	
Race					N/A
African American	40	100	0	–	
Hispanic	0	–	12	30	
Asian	0	–	8	20	
Caucasian	0	–	4	10	
Mixed/Other	0	–	16	40	
Maternal vitamin D supplementation					NS
<400 IU/day	7	17.5	11	27.5	
≥400 IU/day	33	82.5	29	72.5	
Feeding					NS
Exclusively breast fed	11	27.5	19	47.5	
Exclusively formula fed	13	32.5	6	15.0	
Mixed	16	40.00	15	37.5	

^1^ Geometric means and 95% confidence intervals are presented for variables requiring log transformation to conform to normal distribution; ^2^ Undetectable samples assigned as 2/3 detection limit.

In multivariate regression modeling, unadjusted infant WBBMC was most strongly predicted by infant weight at the time of the scan (*p* < 0.0001), while either PTH or BALP contributed modestly but significantly to the model (*p* = 0.006 and *p* = 0.03 respectively). The total *R*^2^ values for the respective models were 0.86 (infant weight and PTH) and 0.85 (infant weight and BALP), with a partial *R*^2^ of 0.79 for infant weight in each model. Multivariate regression modeling of weight-adjusted WBBMC yielded a simple correlation with PTH as described with *R*^2^ = 0.08. Season, maternal age, race, pre-gestational BMI, vitamin D intake, and vitamin D status and infant age at the time of the scan, gestational age at birth, feeding pattern (breast fed, formula fed, or mixed) and vitamin D status did not remain in the final predictive models.

## 5. Discussion

While BALP and PTH, but not 25(OH)D, remaining statistically significant predictors of neonatal WBBMC in multivariate models, infant weight contributed 92–93% of the total *R*^2^ values. This finding corroborates previous indications that body weight is the strongest determinant of WBBMC in infants [19]. After adjusting WBBMC for weight (WBBMC/kg^1.3), PTH remained a weakly significant predictor of adjusted WBBMC in multivariate regression modeling. 

Unadjusted infant WBBMC did not differ significantly between African American and non-African American infants despite higher mean cord PTH and BALP and lower mean cord 25(OH)D in African Americans. The difference in size-adjusted infant WBBMC between African Americans and non-African Americans approached significance (*p* = 0.06), suggesting the possibility of Type I error due to limited sample size. Polymorphisms in the vitamin D receptor gene may contribute to racial disparities in fetal growth and bone mineralization [20,21], although additional research is warranted in this area.

None of the measured indices differed significantly in the maternal or cord blood of women who did or did not take daily prenatal vitamins containing 400 IU vitamin D. The Food and Nutrition Board recommendation for vitamin D intake by pregnant women was raised in 2010 from 200 to 600 IU/day [22]. In the present study, total maternal daily vitamin D intake >600 IU/day was associated with significantly higher cord, and a trend towards higher maternal, serum 25(OH)D at delivery. 

During gestation, active transport of calcium across the placenta maintains a hypercalcemic state in the fetus and PTH is suppressed [6]. In accordance with this model, we found a large proportion of undetectable PTH concentrations (<3 pg/mL, 37.5%) in cord blood samples. Data from a PTH-null mouse model suggests that PTH is essential for fetal cartilage matrix mineralization [9]. Based on the inverse correlation between cord PTH and neonatal WBBMC in our study, however, it is plausible that above its optimal range PTH impedes mineralization. Gopalakrishnan *et al.* have demonstrated *in vitro* PTH-mediated induction of mRNA expression of matrix Gla protein and osteopontin [23,24], two inhibitors of biomineralization [25].

Cord BALP, like cord PTH, was significantly inversely correlated with both cord 25(OH)D and neonatal WBBMC. An association between elevated BALP and impaired bone mineralization as measured by DXA has been shown in pre-term infants [12], anorexic girls [11], and adults [13]. Bone biopsies conducted in children with rickets have shown increased amounts of unmineralized osteoid and prolonged mineralization lag time [26]. It is likely that the elevation in BALP observed in vitamin D deficiency is due to increased release from osteoblasts accumulated in unmineralized bone matrix. Results from the present study provide support for this mechanism in fetal bone development. 

Limitations of this study were the possibility of Type I error due to limited sample size and the fact that additional biomarkers of bone turnover, including those specific to resorption, were not incorporated in analysis. Further research is necessary to consider the behavior of other biomarkers in relationship to vitamin D status and their potential contribution to prediction of neonatal WBBMC. 

## 6. Conclusion

This study provides evidence of associations between feto-maternal vitamin D status, cord PTH and BALP, and neonatal WBBMC, though neither cord nor maternal 25(OH)D were directly correlated with WBBMC. Cord PTH and BALP were statistically but not clinically significant predictors of neonatal WBBMC, which was most strongly predicted by infant weight. Because vitamin D inadequacy during gestation and its impacts on offspring bone integrity are of public health concern, further studies are warranted to investigate the complex mechanisms linking *in utero* vitamin D status to fetal bone development. 
